# Identification of a Novel homozygous Splice-Site Deletion in *KCTD7* Gene Associated with Progressive Myoclonic Epilepsy

**DOI:** 10.12669/pjms.42.9.16091

**Published:** 2026-09

**Authors:** Shatha Alharazy, Peter Natesan Pushparaj, Rose Jelani, Fehmida Bibi, Osama Yousef Muthaffar, Muhammad Imran Naseer

**Affiliations:** 1 Shatha Alharazy Department of Physiology, Faculty of Medicine, King Abdulaziz University, Jeddah 21589, Saudi Arabia; 2 Peter Natesan Pushparaj Institute of Genomic Medicine Sciences (IGMS), King Abdulaziz University, Jeddah 21589, Saudi Arabia; 3 Rose Jelani Institute of Genomic Medicine Sciences (IGMS), King Abdulaziz University, Jeddah 21589, Saudi Arabia; 4 Fehmida Bibi Special Infectious Agents Unit–BSL3, King Fahd Medical Research Centre, King Abdulaziz University, Jeddah 21589, Saudi Arabia; 5 Osama Yousef Muthaffar Department of Pediatrics, Faculty of Medicine, King Abdulaziz University, Jeddah 21589, Saudi Arabia; 6 Muhammad Imran Naseer Institute of Genomic Medicine Sciences (IGMS), King Abdulaziz University, Jeddah 21589, Saudi Arabia

**Keywords:** Epilepsy, KCTD7, Progressive myoclonic epilepsy, WES, Saudi Arabia

## Abstract

**Objective:**

To study the Progressive myoclonic epilepsies (PME) that is genetic disorders resulting from mutations in different genes, all characterized by the early onset of myoclonic seizures, and cognitive decline. The Potassium Channel Tetramerization Domain Containing seven (*KCTD7)* gene encodes for the BRC (broad complex), ttk (tramtrack), and bab/pox virus, and zinc finger domain, containing KCTD7 protein.

**Methodology:**

The study was done in the Center of Excellence in Genomic Medicine and Research (CEGMR). The affected patient, a three-year-old Saudi female born to consanguineous parents, she underwent laboratory tests, EEG assessments, and Whole-Exome Sequencing (WES).

**Results:**

Our results showed a novel five base pair deletion that was detected in homozygous state in the *KCTD7* gene. Both the unaffected parents showed heterozygous mutation of the *KCTD7* gene. Segregation analysis via Sanger sequencing supported the existence of the homozygous splice donor variant in NM_153033.4:c.493+2_493+6delTGA of *KCTD7* gene in the patient.

**Conclusion:**

The mutation is not reported in the literature yet; the particular phenotype that was observed in our patient is comparable to the ones that are described in the *KCTD7* related pathologies, combined with segregation analysis indicating both parents carried the variant heterogeneously present is a strong indication that the identified mutation consists of probably pathogenic mutation. This finding will further increase our insight of the genetic basis of PMEs and role of *KCTD7* gene mutations in Saudi population.

## INTRODUCTION

Progressive Myoclonic Epilepsies, or PME, are actually a group of several disorders that predominantly present with seizures and myoclonus or sudden muscle jerks, it is commonly accompanied by cognitive impairments, such as dementia, and cerebellar involvement manifesting as ataxia and dysarthria.^1^,^2^ These symptoms progress overtime, declining to differing levels of disability, and sometimes early death.

The different types of PME are categorized based on the specific gene affected, next generation sequencing techniques have been a huge advantage in advancing the diagnostic methodology for this disorder. Amongst the most common mutations are biallelic mutations of the cystatin B gene (CSTB), causing Progressive Myoclonic Epilepsy Type-1 (EPM1) or Unverricht-Lundborg disease (ULD).^2^,^3^ Myoclonus is one of the most debilitating symptoms in ULD often managed by polytheraputic intervention targeting the generalized tonic-clonic seizures (GTC) and myoclonic symptoms. Another common mutation is triggered by biallelic variants in necessary glycogen metabolic enzymes Laforin, which is encoded by EPM2A, and malin encrypted by EPM2B, causing a deadly type of PME called Lafora disease.^4^ Its severity comes from the accumulation of glycogen precipitates or polyglucosan bodies, termed Lafora bodies, that drive a neuroinflammatory response.

A less common but severe genetic form of PME is caused by the biallelic loss of function of the *KCTD7* gene causing PME Type-3.^4^ The *KCTD7* gene encodes for the BRC (broad complex), ttk (tramtrack), and bab/pox virus, and zinc finger domain, containing *KCTD7* protein. *KCTD7* is a highly conserved protein that includes 289 amino acids.^5^ Whilst its exact mechanism is still being investigated. *KCTD7* gene is mainly expressed in brain hippocampal neurons and Purkinje cells and it appears to influence cell membrane potentials through an interaction with potassium currents^5^-^8^ as well as several integral roles in neuronal autophagy^8^,^9^ and regulating neuronal glutamine transporters.^7^,^8^

Further explain the progression to autism or schizophrenia in some *KCTD7* ambulatory patients.^8^ A role in proteostasis which can cause the accumulation of undegraded proteins, thus contributing to progressive diseases.^8^,^9^ *KCTD7*-related PME was first identified in 2007 in a large consanguineous Moroccan family, where three female patients presented with early-onset seizures.^10^ Since then, numerous cases with similar clinical presentations have been reported. According to a 2023 systematic review of 42 documented cases, parental consanguinity was observed in almost 50% of patients with this form of PME^11^ (Yoganathan et al., 2024). They reported rare mutation, and observed phenotype includes early onset drug resistant epilepsy with obvious myoclonic seizures and GTCs with subsequent neuroregression almost all cases of this type regress to be partially or completely dependent in their daily activities.^10^,^11^

Multiple variants of *KCTD7* gene mutations have been reported for this type from all over the world such as India representing the largest proportion of reported cases (36.59%), followed by Turkey (29.3%) and only (4.9%) reported cases in Saudi Arabia.

Here we report the presence of a novel variant leading to a homozygous mutation of *KCTD7* gene in a consanguineous family. Where we used WES to identify a heterozygous mutation of the *KCTD7* gene in both unaffected parents leading to a homozygous five base pair deletion in the affected member, who showed PME symptoms at three years of age.

## METHODOLOGY

Blood samples of the patient and parents were taken after the written informed consent was obtained from the parents.. Using the blood samples, DNA extraction process followed as done in the previous studies.^12^,^13^

### Ethical approval: I

t was obtained (Ref# 013-CEGMR-1-ETH, dated: May 1, 2013) from the local ethical committee in the Institute of Genomic Medicine Sciences, King Abdulaziz University Jeddah. The collection of the method and the experimental method were performed according to the international guidelines in Declaration of Helsinki 2013

### Case analysis:

The patient (IV-1) was a three-year-old Saudi female, sibling to one unaffected male from consanguineous parents (Fig.1A), At time of diagnosis, the patient presented myoclonic status epilepticus for two months, myoclonus jerks were occurring between periods extending over two weeks. Patient underwent a number of clinical assessments: pedigree analysis, laboratory data analysis, including a blood test, Magnetic resonance imaging (MRI) and a Sleep electroencephalogram (EEG) etc.

**Fig.1A:**
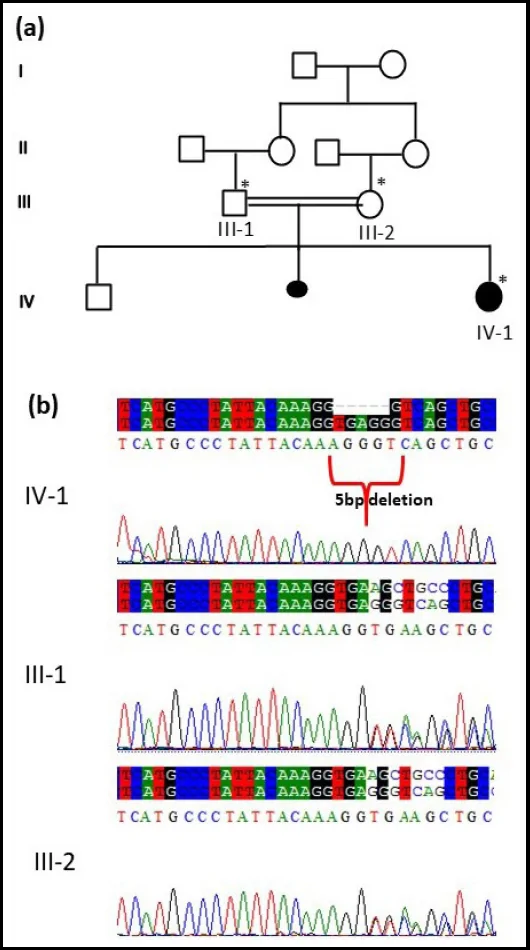
Family Pedigree Chart. affected patient (IV-1) is a daughter to consanguineous parents (III-1, III-2). Fig.1B: Sanger sequencing-based segregation analysis. Variant Description: The proband having undergone WES revealed a homozygous splice site deletion (c.493+2493 +6delTGA) in gene KCTD7 (OMIM 611725). OMIM Phenotype: The segregation analysis conducted by Sanger-sequencing validated the co-occurrence of the c.493+2_493+6delTGA variant of the KCTD7 gene in the patient (IV-1) as well as a heterozygous variant in an unaffected carrier father (III-1) and mother (III-2), which supports the pathogenicity of the mutation and the predicted mechanism of inheritance.

### Laboratory assessment:

MRI was conducted where axial and coronal T2 weighted fast spin echo images, Axial FLAIR, diffusion weighted images and ADC map, sagittal T1 weighted bfast spin echo images without intraveneous contrast injection were taken, no previous MRI studies are available for comparison. A Sleep EEG was done under standard condition using different montages.

### Genetic analysis:

### Whole exome sequencing:

Whole exome sequencing of the patient (IV-2) was conducted with the Illumina HiSeq 2000/2500 (San Diego, CA, United States). The Agilent SureSelect Target Enrichment Kit (Capture Kit, SureSelect v.6, Santa Clara, CA, United States) was used for sampling. The Illumina HiSeq 2000/2500 system was used to perform the library sequencing. Bioinformatics studies were then used to find the variant in the *KCTD7* gene. FASTQ format raw reads were obtained using whole exome sequencing. We differentiated Insertion/deletion and copy number variation with the help of SAMtools:

- The raw data FASTQ files were processed to perform BWA Aligner.
- The VCF file is a total of 111,950 variants. The variants were elucidated by using different parameters: genomic position, quality, protein effect, frequency and pathogenicity. Sequencing was compared to the hg19 human reference assembly (National Center for Biotechnology Information assembly GRCh37).
- The extracted data were cross-linked with dbSNP, population-based databases, including the Genome Aggregation Database (gnomAD), the Exome Variant Server (ESP), and the 1000 Genomes Project, and disease-centered databases, including ClinVar (which contains statements about the clinical relevance of human variants and their phenotype association) and the OMIM (noting human genes and genetic conditions and a selection of disease-related variants) and the Human Gene Mutation Database (HGMD), which includes variant annotation assertions in American College of Medical Genetics and Genomics and the Association of Molecular Pathology (ACMG/AMP). According to the latest guidance (ACMG/AMP) the reported variant was deemed to be of uncertainty relevance.

Variant prioritization was performed through a stepwise filtering approach. Following variant calling and annotation, variants with low sequencing quality were excluded. Common variants (minor allele frequency >1%) present in gnomAD, ExAC, ESP, and the 1000 Genomes Project were removed. The remaining rare variants were filtered based on predicted functional impact, retaining nonsynonymous, frameshift, nonsense, splice-site, and coding indel variants. Given the consanguineous pedigree and presumed autosomal recessive inheritance, homozygous variants were prioritized. Candidate variants were subsequently evaluated using ClinVar, OMIM, HGMD, SIFT, PolyPhen-2, MutationTaster, and phenotype-gene correlation analyses. The *KCTD7* splice-site variant was selected as the most likely disease-causing candidate based on its rarity, predicted functional impact, segregation pattern, and established association with progressive myoclonic epilepsy.

### Sanger sequencing:

The patient and the parents underwent Sanger sequencing to ascertain the availability of the variation observed in the *KCTD7* gene. The extracted data were in a form of the AB1 sequence trace. All of the sequence trace files were aligned to the reference sequence of the corresponding sequence using the BioEdit software. Primers for PCR amplification of *KCTD7* were used such as the forward primer sequence 5’ GACCCTCTTTCCTTCCTGCT 3’ and the reverence primer sequence 5’ TTTGCTAACTGTCATAGGCCA 3’. PCR sequencing was carried out by chain termination reaction. The target variant was easily confirmed using electropherograms obtained from the ABI genetic analyzer. Additionally, 100 controls were used to rule out this mutation in normal population.

## RESULTS

### Clinical history:

The Saudi consanguineous parents had an affected female daughter (Fig.1A). The patient was born with a caesarean section at full term, after an uneventful pregnancy, and had a history of ataxic gait since she was one year of age, as well as dysphagia, delayed speech and motor skills accompanied by regression of milestones. At three years of age, the family reported seizures, abnormal hand and leg tremors, as well as myoclonic jerk at sleep. Physical exam showed signs of upper motor neuron lesion, initial sleep EEG was conducted two months prior to final diagnosis, which showed a background consisting of generalized 4-6 HZ activity of moderate amplitude, symmetrical and synchronous. Generalize fast activity 12-14 HZ of no epileptiform discharges, stage II sleep features of sleep spindles and vertex waves, electrocardiogram of sinus showed regular rhythm. The report concluded in abnormal EEG due to the slow background and right to left generalized predominately frontal 2.2.5 HZ spike and wave. The patient initially was diagnosed with primary cerebral palsy and was started on Keppra, although the regression of milestones showed a need for a whole exome sequencing to rule out genetic origin of seizures.

### Laboratory evaluation:

Blood test results showed normal levels of total cholesterol, triglycerides, alpha feto protein and low-density lipoprotein. But slightly low levels of high-density lipoprotein (52.2 mg/dl) were noted. Sleep electroencephalogram (EEG) (Fig.2) revealed a background which is dominated by theta waves at frequency of four Hertz per second. During recording there is epileptogenic discharge seen in the form of recurrent burst of generalized spike and low wave which is continuous throughout the tracing. Therefore, the report showed epileptogenic discharge seen in the form of recurrent burst of generalized spike and slow wave, consistent with generalized epilepsy.

**Fig.2:**
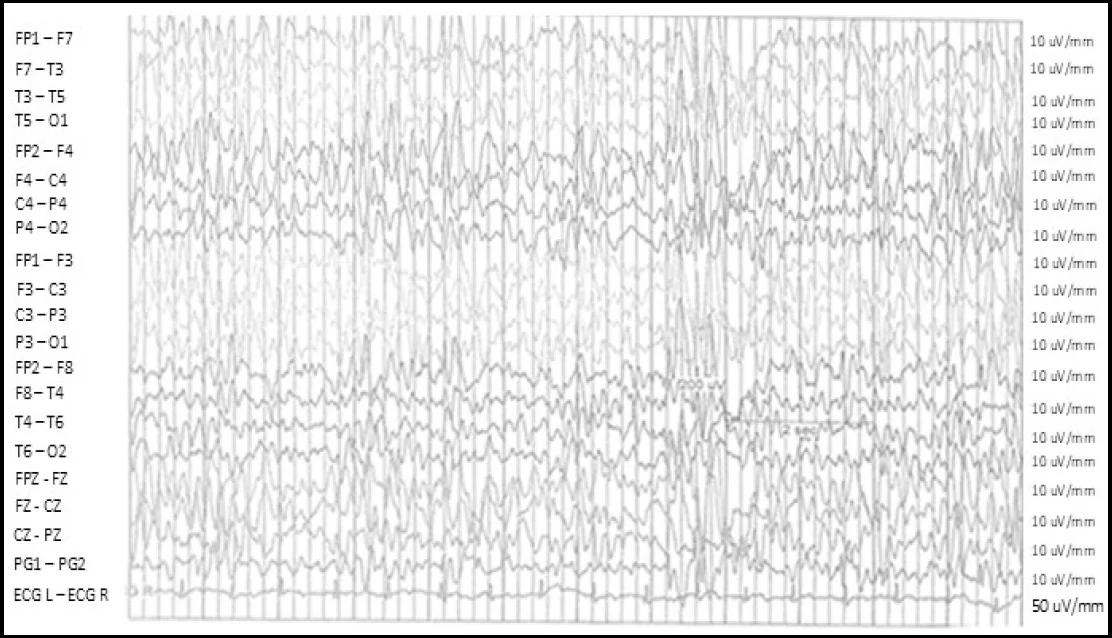
Sleep electroencephalogram (EEG). Sample of our patient, showing slow backgroundand right to left generalized predominantly frontal 2-2.5 HZ spike and waves.

### MRI Findings:

Magnetic resonance imaging (MRI) results showed normal features of the brain with no previous MRI for comparison. the MRI showed normal grey/white matter differentiation of both cerebral hemispheres. The magnetic resonance imaging showed normal morphology of basal ganglia, thalami, internal, and external capsule on both sides; there was homogenization of the intensity of signal on periventricular white matter and the centrum semi-ovale. No focal or diffuse cerebral abnormal intensities. No mass effect or midline shift. Normal size and configurations of the infra and supra tentorial ventricular systems. Normal subarachnoid spaces including cisterns, fissures and sulci. No intra or extra axial collections. Normal patency of the main venous sinuses. Normal caliber and course of the major cerebral arteries with signal void preserved on all sequences (patent) Normal configuration of the optic nerves, optic chiasm, pituitary gland and its stalk. Moreover, normal features of the vermis, cerebellar hemispheres, peduncles and brain stem with no abnormal intensities was seen. Normal MRI features of the cerebellopontine angles and internal auditory canal.

### Genetic analysis:

WES revealed a new splice site mutation (c.493+2_493+6delTGA) at exon- intron boundary occurring at *KCTD7* gene. Sanger sequencing, a screening of both parents, of this variant showed it to be valid. The patient was homozygous in relation to this variant, but the parents were both heterozygous. Thus, confirming the autosomal recessive inheritance of the variation identified (Fig.1B). To the best of our knowledge, this variant has not been published before in literature. Novel variant in gnomAD exomes and 1000 genomes. The TraP score of the position 2-44206963-A-T was calculated to be 0.903, which suggests a potentially detrimental effect.

Furthermore, filtering of sequenced variants was done afterwards to retain only those that were considered to be novel or rare (minor allele frequency ≤0.01%). The retained variants were further penetrated into the category of functionally predicted damaging through SIFT and PolyPhen pathogenicity analyses. The variant was also found to be a disease-causing one by Mutation Taster. Moreover, the variant was not present in the ExAC or the 1000G databases. We also reported the *KCTD7* pathogenic and likely variation enlisted in the ClinVar database as shown in (Table-I). This information will give the reader an updated overview of the *KCTD7* related variations causing PME. The variant classification criteria of the ACMG, i.e. PVS1, PM2, indicate that this variant identified in the *KCTD7* gene is probably pathogenic. The separation of the variant also supports its bad properties. Many new novel disease-causing variants related to the *KCTD7* gene are increasingly getting recognized as shown in (Table-I). The variant NM_153033.5(*KCTD7*):c.493+2_493+6del was not detected in population databases including gnomAD, ExAC, and the 1000 Genomes Project. In addition, a search of ClinVar and HGMD revealed no prior reports of this specific variant, indicating that it is novel. The absence of the variant from large population datasets and disease-specific databases provides moderate evidence for pathogenicity (PM2) according to ACMG/AMP guidelines.

**Table-I:** KCTD7 single nucleotide polymorphisms and small indels (≤10 base pairs) reported to cause progressive myoclonus epilepsies in the literature, as documented pathogenic and likely pathogenic in ClinVar database known so far.

No	Origin	Zygosity	Variant	Protein	ACMG interpretation	References
1	Turkey	Homozygous	NM_153033.5: c.130dup	(p. Leu44fs)	Pathogenic	6
2	Turkey	Homozygous	NM_153033.5:c.139C>T	(p. Gln47Ter)	Pathogenic	6
3	United States	Compound Heterozygote	NM_153033.5: c.145-2A>G, c.190A>G	- (p.T64A)	Likely pathogenic	18
4	India	Homozygous	NM_153033.5:c.205C>G	(p.Leu6 9Val)	Pathogenic	15
5	Turkey	Homozygous	NM_153033.5:c.818A>T	p.Asn273Ile	pathogenic	6
6	Turkey	Homozygous	NM_153033.5: c.280C>T	p.Arg94Trp	Pathogenic	6
7	Turkey	Compound Heterozygote	NM_153033.5(KCTD7): (c.343G>T, and c.818A>T)	(p.Asp115Tyr), p.Asn273Ile	pathogenic	6
8	Morocco	Homozygous	NM_153033.5(KCTD7):c.295C>T	(p.Arg99Ter)	Pathogenic	10
9	Turkey	Homozygous	NM_153033.5(KCTD7):c.314+1G>A		Likely pathogenic	6
10	Pakistan	Homozygous	NM_153033.5(KCTD7):c.322C>A	(p.Leu108Met)	Pathogenic	6
11	Turkey	Homozygous	NM_153033.5(KCTD7):c.331del	(p.Leu111fs)	Pathogenic	6
12	Turkey	Compound Heterozygous	NM_153033.5(KCTD7): c.594delC, c.818A>T	p.Ile199SerfsX74, p.Asn273Ile	Pathogenic	6
13	Turkey	Homozygous	NM_153033.5(KCTD7): c.594delC	p.Ile199SerfsX74	Pathogenic	6
14	Turkey	Homozygous	NM_153033.5(KCTD7):c.367C>T	(p.Arg123Ter)	Pathogenic	6
15	Turkey	Homozygous	NM_153033.5(KCTD7):c.388C>T	(p.Gln130Ter)	Pathogenic	6
16	India	Homozygous	NM_153033.5(KCTD7):c.458G>C	(p.Arg153Pro)	Likely pathogenic	15
17	United States	Homozygous	NM_153033.5(KCTD7):c.514G>T	(p.Glu172Ter)	Pathogenic	7,19
18	China	Compound heterozygous	NM_153033.5(KCTD7): c.440T>C, c.520G>A	p.Leu147Pro,(p.Ala174Thr)	pathogenic	20
19	Mexico	Homozygous	NM_153033.5(KCTD7):c.550C>T	(p.Arg184Cys)	Pathogenic	21
20	Saudi Arabia	Homozygous	NM_153033.5(KCTD7):c.696del	(p.Phe232fs)	Pathogenic	7
21	Turkey	Homozygous	NM_153033.5(KCTD7):c.818A>T	(p.Asn273Ile)	Pathogenic	6
22	Canada	Homozygous	NM_153033.4: c.827A>G,	p.Tyr276Cys	Pathogenic	19
23	Oman	Homozygous	NM_153033.5: c.335G>A	p.Arg112His	Likely pathogenic	22
24	India	Homozygous	NM_153033.5: c.208C>T, p	(Arg70Trp)	Likely pathogenic	9
25	India	Homozygous	NM_153033.5: c.250C>T,	p.(Arg84Trp)	Likely pathogenic	23
26	USA	Compound heterozygous	NM_153033.5: 1. c.541C>T, 2. c.685G>T,	p.(Arg181Trp), p.(Asp229Tyr)	Likely pathogenic,	9
27	USA	Homozygous	NM_153033.5: c.334C>T	p.(Arg112Cys)	pathogenic	9
28	India	Homozygous	NM_153033.5: c.493C>T	p.(Arg165Trp)	Likely pathogenic	24
29	India	Homozygous	NM_153033.5: c.533C>T,	p.(Ala178Val)	Likely pathogenic	24
30	India	Homozygous	NM_153033.5: c.493 + 1G>A	5′ splice site–intron 3	Likely pathogenic	11
31	India	Homozygous	NM_153033.5: c.367C>T	p.(Arg123Ter)	Likely pathogenic	11
32	India	Homozygous	NM_153033.5: c.281G>T	p.(Arg94Leu)	Likely pathogenic	11
33	India	Homozygous	NM_153033.5: c.551G>A	p.(Arg184His)	Likely pathogenic	11
34	India	Homozygous	NM_153033.5: c.281G>A	p.(Arg94Gln)	Likely pathogenic	11
35	India	Homozygous	NM_153033.5: c.754C>T	p.(Leu252Phe)	Likely pathogenic	11
36	Canada	Homozygous	NM_153033.5: c.386C>A	p.(Ala129Asp)	Likely pathogenic	11
37	India	Homozygous	NM_153033.5: c.280C>G	p.(Arg94Gly)	Likely pathogenic	11
38	India	Homozygous	NM_153033.5: c.54G>A	p.(Met18Ile)	Likely pathogenic	11
39	India	Compound heterozygous	NM_153033.5: c.207_214delGCGGTGCT, c.496C>G	p.Cys71Argfs*130 p.(His166Asp)	Likely pathogenic	11
40	India	Homozygous	NM_153033.5: c.458G>T	p.(Arg153Leu)	Likely pathogenic	11
41	Saudi Arabia	Homozygous	NM_153033.4:c.493+2_493+6delTGA	-	Likely Pathogenic	Current Study

### Sanger sequencing validation:

The results from the WES were further validated using Sanger sequencing analysis and this confirmed the presence of a novel splice-site variant (c.493+2_493+6delTGA) at the exon-intron boundary in the *KCTD7* gene causing progressive myoclonic epilepsy in a Saudi proband IV-4 as shown in Fig.1B.

## DISCUSSION

In this study we are reporting a novel five base pair deletion that was detected in homozygous state in the *KCTD7* gene from the Saudi patient with epilepsy. Progressive myoclonic epilepsies (PME) are rare genetic disorders largely presenting with symptoms like myoclonic seizures and neuroregression.^1^ As the name suggests, these disorders progress over time, effecting patients’ daily lives and preventing them from independency and sometimes causing an early end to their life.^2^ PME Type-3 (PME3) is caused by mutations in the *KCTD7* gene.^4^ PME3 are specifically a severe form of this disorder as they are usually drug resistant, and due to their rareness, there is a lack of understanding when it comes to the mechanism and exact effects of *KCTD7* gene mutations.

Up to date there has been less than one hundred reported cases of *KCTD7* related PME’s worldwide. In this study, we report a novel homozygous mutation c.493+2_493+6delTGA in *KCTD7* in a 3-year-old Saudi girl from a consanguineous family. WES analysis for the patient and parents showed a homozygous mutation in the patient and heterozygous in the parents.

The patients’ phenotype is consistent with what’s been previously reported in the literature about PME,^2^,^14^,^15^ with the presenting symptoms being early-onset seizures and regression of milestones, In our patient the main seizure type consisted of reoccurring multifocal myoclonic seizures affecting her hands and legs. The average reported age of disease onset, when symptoms first start to appear, is usually from five months to three years,^15^ in our current case, the patients’ seizures were reported at three years of age, falling under the expected timeline. Moreover, the patient also exhibited an autosomal recessive pattern as the previous literature describes PME cases^6^,^10^,^15^ where both parents presented with a heterozygous while affected daughter had the homozygous alleles.

Laboratory evaluation for our patient showed EEG abnormalities, predominantly characterized by highly frequent generalized multifocal spike and wave discharges, consistent with the usual presenting phenotype.^1^,^2^,^11^ The neuroimaging evidence of the MRI in patients with progressive myoclonic epilepsy (PME) lacks any regular patterns of diagnostics and can remain unexplainable in the early stages of the disease^11^, Yet, with the further progression of the condition, the cerebral or cerebellar atrophy as well as the white matter changes might become more pronounced.^16^,^17^ In the current instances, MRI of the brain was mentioned to be normal.

The highly specific phenotype observed in our patient with *KCTD7*-related diseases (IV-1), combined with segregation analysis indicating both parents had heterogenous variant clearly suggest that variant identified is pathogenic.

### Limitations:

A limitation of this study is the absence of functional RNA-based assays and in silico splice prediction analyses (e.g., SpliceAI or MaxEntScan) to directly evaluate the effect of the *KCTD7* splice-site variant on transcript processing.

## CONCLUSION

In this study, we report a novel likely pathogenic variant (c.493+2_493+6delTGA) of the *KCTD7* gene in a three-years-old daughter from Saudi consanguineous parents, who were carries of a heterozygous variant. The patient suffered progressive myoclonic seizures, developmental delay, and neuroregression. WES analysis and sanger sequencing techniques helped us identify and validate this *KCTD7* variant, adding to the Saudi Arabian existing dataset of PME genetic variants, and advancing our understanding of *KCTD7* related PMEs, contributing to developing better future genetic therapy targets.

### Recommendations:

Future studies incorporating transcript analysis and functional splicing assays will be important to confirm the predicted loss-of-function mechanism. In the present family, the autosomal recessive inheritance pattern indicates a 25% recurrence risk for future pregnancies. Therefore, carrier testing, prenatal diagnosis, and preimplantation genetic testing may be considered for future reproductive planning. Furthermore, because consanguineous marriages remain common in many Middle Eastern populations, the identification of novel *KCTD7* variants contributes to regional variant databases and may support future carrier-screening and precision medicine initiatives aimed at improving the diagnosis and prevention of rare inherited neurological disorders. The identification of a likely pathogenic *KCTD7* variant has important clinical implications. A definitive molecular diagnosis facilitates accurate genetic counseling, informs recurrence risk assessment, and enables targeted testing of at-risk family members.
